# Causal associations between leisure sedentary behaviors and sleep status with frailty: insight from Mendelian randomization study

**DOI:** 10.1186/s12877-024-04758-z

**Published:** 2024-02-17

**Authors:** Chuang Li, Na Li, Hailong Huang, Yangyang Li, Yanyan Zhuang

**Affiliations:** 1 Department of Obstetrics & Gynecology, Shengjing Hospital of China Medical University, Shenyang, China; 2 Key Laboratory of Maternal-Fetal Medicine of Liaoning Province, Shenyang, China

**Keywords:** Genetic correlation, Sleep duration, Time spent watching television, Daytime napping, Causality

## Abstract

**Background:**

Observational studies have suggested that sedentary behaviors and sleep status are associated with frailty. However, it remains unclear whether these associations are causal.

**Methods:**

Using summary statistics from genome-wide association studies, we evaluated the causal effect of modifiable risk factors, including leisure sedentary behaviors and sleep status on the frailty index (FI) using two-sample univariable and multivariable Mendelian randomization (MR) analyses. Genetic correlations were tested between the correlated traits.

**Results:**

We identified potential causal associations between the time spent watching television (*β* = 0.26, 95% confidence interval [CI]: 0.21–0.31, *P* = 3.98e-25), sleep duration (*β* = -0.18, 95%CI: -0.26, -0.10; *P* = 6.04e-06), and daytime napping (*β* = 0.29, 95%CI: 0.18–0.41, *P* = 2.68e-07) and the FI based on the inverse-variance-weighted method. The estimates were consistent across robust and multivariate MR analyses. Linkage disequilibrium score regression detected a genetic correlation between the time spent watching television (Rg = 0.43, *P* = 6.46e-48), sleep duration (Rg = -0.20, *P* = 5.29e-10), and daytime napping (Rg = 0.25, *P* = 3.34e-21) and the FI.

**Conclusions:**

Genetic predispositions to time spent watching television and daytime napping were positively associated with the FI, while sleep duration was negatively associated with the FI. Our findings offer key insights into factors influencing biological aging and suggest areas for interventions to promote healthy aging and slow down the aging process.

**Supplementary Information:**

The online version contains supplementary material available at 10.1186/s12877-024-04758-z.

## Background

Frailty is a complex clinical condition that is intricately linked to the aging process and is accompanied by a decline in physiological function across multiple organ systems, with increased susceptibility to stressors [1, 2]. As the global population ages, the prevalence of frailty increases proportionately [3]. Beyond its role as a noteworthy predictor of mortality among older individuals, frailty is implicated in a spectrum of adverse health outcomes, including falls, delirium, and disability [4–6]. In recent years, frailty has garnered considerable attention as a public health concern and tremendous global health challenge.

The roles of sedentary behavior and sleep status in the etiology of frailty have been extensively investigated [7–10]. However, owing to limitations in the quality of available evidence, as well as the presence of potential reverse causality and residual confounding factors, observational studies have been unable to establish causal associations. To address this issue, randomized controlled trials (RCTs) have been conducted to elucidate cause-and-effect relationships [11]. Nonetheless, RCTs are often resource-intensive in terms of finances and manpower, and certain interventions may not be feasible or approved for assessment. In this context, Mendelian randomization (MR) is effective for estimating the causal effects of exposures on outcomes [12, 13]. By employing genetic variants that are robustly associated with the exposure of interest as instrumental variables (IVs) randomly assigned at conception, MR studies mitigate the confounding and reverse causality biases that are inherent in conventional observational studies [14, 15].

We conducted univariable and multivariable MR analyses to examine potential independent causal effects of leisure sedentary behaviors and sleep status on the frailty index (FI). Linkage disequilibrium score (LDSC) regression was used to investigate the genetic correlation between these causal traits.

## Methods

### Data source of exposure

Watching television, computer usage, and driving were identified as three distinct categories of sedentary behaviors [16]. To ascertain the extent of sedentary time, the participants were asked a set of three questions during their initial visit. These inquiries encompassed the following: “On a typical day, how many hours do you spend watching television?,” “In a typical day, how many hours do you spend using the computer? (Do not include using a computer at work),” and “On a typical day, how many hours do you spend driving?.” The daily duration of these sedentary behaviors served as a measure of exposure assessment. The study population comprised 408,815 individuals of European ancestry enrolled from the UK Biobank. The participants had an average age of 57.4 (± 8.0) years, and females constituted 54.3% of the cohort. Mean daily reported leisure television watching was 2.8 h (± 1.5), leisure computer use was 1.0 h (± 1.2) and driving was 0.9 h (± 1.0).

We acquired genetic instruments for sleep duration through a genome-wide association study (GWAS) conducted using the UK Biobank dataset [17]. Within the UK Biobank, the sleep duration assessment involved a specific inquiry: “How many hours of sleep do you get during every twenty-four hours (including naps)?” The results were scaled to per-hour increases in sleep duration. The study encompassed a cohort of 446,118 individuals with European ancestry, characterized by a mean age of 57.3 (± 8.0) years. Notably, women constituted 54.1% of the participant group. The analysis revealed mean self-reported habitual sleep duration was 7.2 h (± 1.1) per 24 h.

Genetic variants associated with snoring were obtained from the UK Biobank population [18]. During the assessment, participants were asked the following question: “Does your partner or a close relative or friend complain about your snoring?” Response options included “Yes,” “No,” “Don’t know,” or “Prefer not to answer.” Individuals who responded with “Don’t know” or “Prefer not to answer” were excluded from the dataset. Snoring data were available in 359,916 unrelated individuals of European descent after quality control. The prevalence of snoring in this sample was 37.3%.

Daytime napping is an uncontrollable sleep pattern. We acquired genetic variants associated with daytime napping from an extensive UK Biobank dataset comprising 452,633 participants [19]. During the assessment, the participants were asked, “Do you take a nap during the day?” Among the UK Biobank population, 38.2% and 5.3% of the respondents answered “sometimes” and “always,” respectively. Moreover, the average ages of the two groups were 58.5 (± 7.8) years and 60.2 (± 7.4) years, with females comprising 50.0% and 33.9% of each respective group.

### Data source of outcome

We identified genetic variants associated with the FI using a GWAS meta-analysis. The GWAS included 164,610 participants from the UK Biobank and 10,616 from TwinGene [20]. The UK Biobank participants consisted of individuals of European descent, aged 60 to 70 years at baseline (mean 64.1, SD 2.8). The cohort included 84,819 females, accounting for 51.3% of the total participants. The TwinGene participants, a separate cohort comprising 10,616 individuals, were Swedish nationals aged 41 to 87 years (mean 58.3, SD 7.9), with 5,577 females (52.5%). The Rockwood FI, which is based on the deficit accumulation model, served as the outcome measure for frailty. Compliance with deficits was categorized using a score of 0 or 1 (with 0 indicating no deficit). The FI for each individual was calculated as the number of deficits divided by 49. A higher FI value indicates a greater degree of frailty. Our findings revealed that the mean proportions of deficits were 0.129 ± 0.075 and 0.121 ± 0.080, in UK Biobank and TwinGene participants, respectively.

The baseline characteristics of participants included in these above GWAS studies were presented in Additional file 2: Table S1.

### Selection criteria for instrumental variables

To ensure the validity of each instrumental variable (IV), three key assumptions must be met: (1) relevance assumption: robust association between the instrument and the exposure; (2) independence assumption: the genetic variant is not linked to confounding factors influencing the exposure-outcome relationship [21]; and (3) exclusion restriction assumption: instruments solely impact the outcome through the exposure variable [22]. To fulfill the first assumption of MR, we identified single nucleotide polymorphisms (SNPs) that achieved genome-wide significance (*P* < 5 × 10^− 8^). From this SNP set, we exclusively retained independent instruments with the most significant *P*-values, considering pairwise linkage disequilibrium (LD) and removing SNPs with r^2^ ≥ 0.001. We further *evaluated* the first assumption by computing the *F*-statistic [23]. Subsequently, we excluded SNPs with an *F*-statistic < 10 to avoid weak IV biases. Genetic variants from diverse studies were combined regarding their effects and palindromic SNPs were excluded. Proxy SNPs (r^2^ > 0.8) were used for instruments absent from the outcome dataset. The MR-Steiger filtering was also used to removes SNPs failing to explain significantly more variance in the exposure than in the outcome. To mitigate the bias arising from horizontal pleiotropy, we performed the Mendelian Randomization Pleiotropy RESidual Sum and Outlier (MR-PRESSO) outlier test, calculated the *P value* for each SNP, and excluded outlier SNPs [24]. The lead SNPs for the genetic instruments of exposure were presented in Additional file 2: Tables S2–S7.

### Univariable Mendelian randomization

We employed the inverse-variance-weighted (IVW) approach as the primary method for estimating causal effects, which allows calculation of the combined effect of all SNPs. Additionally, to ensure reliability and stability of the results, sensitivity analyses were conducted using MR-Egger [25], weighted median [26], and MR-PRESSO [24]. Heterogeneity in the main IVW and MR-Egger estimates was assessed by quantifying Cochran’s Q statistic, where *P* > 0.1 indicated the absence of heterogeneity among the instrumental variables, enabling the disregard of its influence on causal effect estimation [27]. For cases in which heterogeneity was detected, the IVW (multiplicative random-effects) approach was used to calculate the effect size. The Egger model intercept was used for statistical assessment of pleiotropy, with a deviation of 0, suggesting the presence of directional pleiotropy [28].

### Multivariable Mendelian randomization

To validate the second core assumption of MR, which entails assessing the absence of associations between the IVs and confounders, we conducted a thorough search of the PhenoScanner database [29]. This search aimed to identify any previously reported significant associations (*P* < 5 × 10^− 8^) between the instrument SNPs and potential confounding factors. Notably, the PhenoScanner search identified associations between the instruments and traits related to obesity, alcohol consumption, and smoking. Consequently, we specifically selected SNPs associated with body mass index (BMI) [30], drinks per week [31], and cigarettes per day [31] from the publicly available GWAS summary statistics to conduct multivariable MR (MVMR) analyses to account for indirect pathways that may introduce correlated pleiotropy. Causal effects were estimated using the MVMR-IVW and MVMR-Egger methods. Heterogeneity among the selected genetic variants was assessed using Cochran’s Q statistic, and MVMR-Egger was employed to identify potential directional pleiotropy.

### LDSC regression analysis

LDSC regression analysis is dependable and effective for detecting the shared genetic frameworks of intricate human characteristics [32]. It enables the estimation of disease heritability and the examination of genetic correlations using GWAS summary data. In the present study, we used GWAS summary data on the identified causal traits to assess their genetic associations. The LD reference panel, obtained from 1000 Genomes and developed by the researchers (source: https://github.com/bulik/ldsc), consisted of the European LD scores utilized in our analysis.

This study followed the Strengthening the Reporting of Observational Studies in Epidemiology Using Mendelian Randomization (STROBE-MR) statement [33] (Additional file 1). All MR analyses were conducted using “TwoSampleMR,” “MRPRESSO,” “MendelianRandomization,” and “MVMR” in R software (version 4.0.2; the R Foundation for Statistical Computing, Vienna, Austria).

## Results

### Associations between leisure sedentary behaviors, sleep status and the FI

**Fig. 1:**
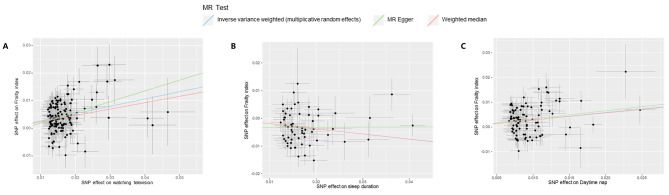
Scatter plot of the causal effect of watching television **(A)**, sleep duration **(B)**, and daytime napping **(C)** on FI using different MR methods

After ensuring quality control, we selected 138 SNPs related to watching television, 442 SNPs related to computer usage, 44 SNPs related driving, 62 SNPs related to sleep duration, 332 SNPs related to snoring, and 93 SNPs related to daytime napping as IVs. The MR study found that the genetic predisposition to spend time watching television (*β* = 0.26, 95% confidence interval [CI]:0.21–0.31, *P* = 3.98e-25) and daytime napping (*β* = 0.29, 95%CI: 0.18–0.41, *P* = 2.68e-07) were positively associated with the FI based on the IVW method, while the sleep duration (*β* = -0.18, 95%CI: -0.26, -0.10; *P* = 6.04e-06) was negatively associated with the FI (Table 1; Fig. 1). All MR results were robust in several sensitivity analyses (Table 1). There was no obvious heterogeneity for the genetic variants of time spent on driving (all *P-*values for Cochran’s Q > 0.1), whereas genetic instrumental variables of other exposures exhibited persistent heterogeneity (Additional file 2: Table S8). All *P-*values for the intercepts of MR-Egger tests were > 0.05 (Additional file 2: Table S9).

**Table 1:** Univariable MR results for the causal associations between leisure sedentary behaviors, sleep status and the FI

Exposure	No of SNPs	F -statistics	IVW		MR-Egger		Weighted median		MR-PRESSO	
			Beta (95%CI)	*P* value	Beta (95%CI)	*P* value	Beta (95%CI)	*P* value	Beta (95%CI)	*P* value
**Leisure sedentary behavior**										
Watching television	138	40.78	0.26 (0.21–0.31)	3.98e-25^*^	0.39 (0.15–0.63)	1.96e-03^*^	0.23 (0.17–0.29)	7.22e-13^*^	0.26 (0.21–0.31)	6.45e-19^*^
Computer usage	42	38.97	0.01 (-0.11–0.14)	0.82	-0.30 (-1.09–0.49)	0.46	0.03 (-0.10–0.16)	0.63	0.01 (-0.11–0.14)	0.82
Driving	4	38.25	0.36 (0.03–0.70)	0.03	2.59 (0.65–4.52)	0.12	0.21 (-0.11–0.53)	0.20	0.36 (0.03–0.70)	0.13
**Sleep status**										
Sleep duration	62	47.86	-0.18 (-0.26 – -0.10)	6.04e-06^*^	0.01 (-0.29–0.30)	0.97	-0.19 (-0.28– -0.10)	6.63e-05^*^	-0.18 (-0.26– -0.10)	2.85e-05^*^
Snoring	32	171.77	0.04 (-0.01–0.08)	0.10	-0.13 (-0.37–0.10)	0.27	0.03 (-0.03–0.08)	0.35	0.04 (-0.01–0.08)	0.11
Daytime napping	94	15.14	0.29 (0.18–0.41)	2.68e-07^*^	0.33 (-0.09–0.75)	0.13	0.28 (0.15–0.41)	1.80e-05^*^	0.29 (0.18–0.41)	1.48e-06^*^

Abbreviation: FI frailty index; MR, Mendelian randomization; IVW, inverse-variance-weighted; MR-PRESSO, Mendelian Randomization Pleiotropy RESidual Sum and Outlier; CI, confidence interval

^*^P value < 0.05

**Table 2:** Summary of the GWAS data used in the MR analyses

Phenotype	Sample size	Ancestry	Consortium/cohort	Year	PMID
**Exposure**					
**Leisure sedentary behavior**					
Watching television	408,815	European	UK Biobank	2020	32,317,632
Computer usage	408,815	European	UK Biobank	2020	32,317,632
Driving	408,815	European	UK Biobank	2020	32,317,632
**Sleep status**					
Sleep duration	446,118	European	UK Biobank	2019	30,846,698
Snoring	359,916	European	UK Biobank	2019	30,804,565
Daytime napping	452,633	European	UK Biobank	2019	33,568,662
**Outcome**					
Frailty index	175,226	European	UK Biobank and TwinGene	2021	34,431,594
**Confounders**					
BMI	681,275	European	GIANT	2018	30,124,842
Alcohol consumption	941,280	European	GSCAN	2019	30,643,251
Smoking heaviness	377,334	European	GSCAN	2019	30,643,251

Abbreviation: MR, Mendelian randomization; BMI, body mass index

Multivariable MR analyses adjusting for confounders provided similar results and also suggested a positively causal effect of time spent on watching television (*β* = 0.27, 95% CI: 0.20–0.35, *P* = 1.19e-12) and daytime napping (*β* = 0.23, 95%CI: 0.10–0.36, *P* = 4.89e-04) and negative causal effect of sleep duration (*β* = -0.18, 95%CI: -0.27, -0.09; *P* = 1.55e-04) on the FI (Table 3). All directions and the statistical significance of the IVW results in MVMR were consistent with those of the MVMR-Egger sensitivity analysis, suggesting a low risk of bias due to horizontal pleiotropy (Table 3; Additional file 2: Table S9). The MVMR heterogeneity test validated sustained heterogeneity across the selected genetic variants (Additional file 2: Table S10).

**Table 3:** Multivariable MR results for the causal association of watching television, sleep duration, and daytime napping with FI

Exposure	Adjustment	Method	Beta	95%CI	*P* value
Watching television	BMI, cigarettes per day, and drinks per week	MVMR-IVW	0.27	0.20–0.35	1.19e-12^*^
		MVMR-Egger	0.27	0.19–0.34	2.44e-12^*^
Sleep duration	BMI, cigarettes per day, and drinks per week	MVMR-IVW	-0.17	-0.27– -0.09	1.55e-04^*^
		MVMR-Egger	-0.17	-0.26– -0.08	2.17e-04^*^
Daytime napping	BMI, cigarettes per day, and drinks per week	MVMR-IVW	0.23	0.10–0.36	4.89e-04^*^
		MVMR-Egger	0.20	0.02–0.38	0.03^*^

Abbreviation: FI, frailty index; MVMR, multivariable Mendelian randomization; IVW, inverse-variance weighted; CI, confidence interval; BMI, body mass index

^*^P value < 0.05

### Genetic correlation between causal traits

We tested the positive genetic correlation between the time spent watching television (Rg = 0.43, *P* = 6.46e-48) and daytime napping (Rg = 0.25, *P* = 3.34e-21) and the FI. LDSC regression analysis revealed a negative genetic correlation between sleep duration (Rg = -0.20, *P* = 5.29e-10) and the FI.

## Discussion

This MR study investigated the potential causal relationships between the leisure sedentary behaviors, sleep status and the FI. We found that time spent watching television, sleep duration, and daytime napping were causally associated with the FI. Multiple sensitivity analyses confirmed the robustness of these causal relationships. MVMR analyses demonstrated independent causal effects of watching television, sleep duration, and daytime napping on the FI, after adjustments for other confounders. We observed a significant genetic correlation between the time spent watching television, sleep duration, daytime napping, and the FI based on the LDSC regression.

Population-based investigations have consistently demonstrated that sedentary behavior contributes to adverse health outcomes. A meta-analysis revealed that reduced sedentary duration is significantly associated with a decreased risk of premature mortality, particularly among middle-aged and older adults, exhibiting a nonlinear dose-response pattern [34]. Considering the findings of our research and those from previous studies, reducing sedentary duration during leisure activities has potential benefits for mitigating the aging process.

Significant causal associations were observed between certain sleep status traits and the FI. Numerous studies have reported a relationship between sleep and aging. A study conducted in a Chinese population comprising 23,847 individuals revealed that maintaining a comprehensive and healthy sleep pattern was positively linked to a reduced risk of worsening frailty and an increased likelihood of improving frailty [10]. Another cross-sectional study utilizing data from the National Health and Aging Trends Study, identified difficulty initiating sleep as an independent risk factor for frailty [35]. Furthermore, a meta-analysis involving 313,651 participants from 20 cohort studies demonstrated that prolonged napping is associated with a higher risk of all-cause mortality [36]. After adjusting for relevant factors, we provide compelling evidence supporting significant causal relationships between sleep duration and daytime napping with the FI.

Our research emphasizes the genetic link between television-viewing time, sleep duration, daytime napping, and the FI. One notable strength of our study is the use of genetic data from a large sample population, which enhanced the reliability of our findings and minimized the impact of confounding factors. Furthermore, the robustness of IVW estimates in this study was supported by multiple MR sensitivity analyses, each incorporating different assumptions regarding genetic pleiotropy. Nevertheless, some limitations of this study should be acknowledged. First, our analysis assumed a linear association between risk factors and outcomes. Although the estimates reflect the presence and direction of the population-averaged causal effect, quantitative interpretations may be misleading if the actual relationship is nonlinear [37]. Second, genetic associations were derived from data of European populations, and caution should be exercised when generalizing these findings to other ethnic groups. Third, the MR analysis estimates were formulated to evaluate the causal impact of long-term exposure on outcomes and, therefore, may not always align precisely with clinical observations. Fourth, while we attempted to strictly adhere to the STROBE-MR guidelines, we were unable to provide all the recommended items because of the restricted information accessible from the utilized database.

## Conclusion

In summary, we utilized MR techniques to provide valuable quantitative data on modifiable risk factors that causally influence the aging process. Understanding the causal effects of these risk factors on frailty holds considerable promise for elucidating the underlying mechanisms of the aging process and establishing potential strategies for preventing age-related diseases and promoting healthy aging. Notably, significant effects were observed for the time spent watching television, sleep duration, and daytime napping. These findings provide crucial insights into the determinants of biological aging and highlight potential areas for intervention to promote healthy longevity and attenuate the rate of biological aging.

### Electronic supplementary material

Below is the link to the electronic supplementary material.

Supplementary Material 1

Supplementary Material 2

## Data Availability

UK Biobank data can be found at http://www.nealelab.is/uk-biobank/. Data from meta-GWASs can be found in the raw publications. The PubMed or database ID of each GWAS is shown in Table 1.

## Abbreviations

MR: Mendelian randomization
UVMR: Univariable Mendelian randomization
MVMR: Multivariable Mendelian randomization
GWAS: Genome-wide association study
IV: Instrumental variable
SNP: Single nucleotide polymorphism
IVW: Inverse variance weighted
MR-PRESSO: MR-pleiotropy residual sum and outliers
OR: Odds ratio
CI: Confidence interval
LD: Linkage disequilibrium
FI: Frailty index
BMI: Body mass index

## References

[1] Hoogendijk EO, Afilalo J, Ensrud KE, Kowal P, Onder G, Fried LP (2019). Frailty: implications for clinical practice and public health. Lancet.

[2] Clegg A, Young J, Iliffe S, Rikkert MO, Rockwood K (2013). Frailty in elderly people. Lancet.

[3] Dent E, Martin FC, Bergman H, Woo J, Romero-Ortuno R, Walston JD (2019). Management of frailty: opportunities, challenges, and future directions. Lancet.

[4] Fried LP, Tangen CM, Walston J, Newman AB, Hirsch C, Gottdiener J, Seeman T, Tracy R, Kop WJ, Burke G (2001). Frailty in older adults: evidence for a phenotype. J Gerontol Biol Sci Med Sci.

[5] Walston J, Hadley EC, Ferrucci L, Guralnik JM, Newman AB, Studenski SA, Ershler WB, Harris T, Fried LP (2006). Research agenda for frailty in older adults: toward a better understanding of physiology and etiology: summary from the American Geriatrics Society/National Institute on Aging Research Conference on Frailty in older adults. J Am Geriatr Soc.

[6] Eeles EM, White SV, O’Mahony SM, Bayer AJ, Hubbard RE (2012). The impact of frailty and delirium on mortality in older inpatients. Age Ageing.

[7] da Silva VD, Tribess S, Meneguci J, Sasaki JE, Garcia-Meneguci CA, Carneiro JAO, Virtuoso JS (2019). Association between frailty and the combination of physical activity level and sedentary behavior in older adults. BMC Public Health.

[8] Lin YK, Chen CY, Cheung DST, Montayre J, Lee CY, Ho MH (2022). The relationship between physical activity trajectories and frailty: a 20-year prospective cohort among community-dwelling older people. BMC Geriatr.

[9] Sun M, Wang L, Wang X, Tong L, Fang J, Wang Y, Yang Y, Li B (2023). Interaction between sleep quality and dietary inflammation on frailty: NHANES 2005–2008. Food Funct.

[10] Zhu Y, Fan J, Lv J, Guo Y, Pei P, Yang L, Chen Y, Du H, Li F, Yang X (2022). Maintaining healthy sleep patterns and frailty transitions: a prospective Chinese study. BMC Med.

[11] Stanley K (2007). Design of randomized controlled trials. Circulation.

[12] Davey Smith G, Hemani G (2014). Mendelian randomization: genetic anchors for causal inference in epidemiological studies. Hum Mol Genet.

[13] Ference BA, Holmes MV, Smith GD. Using mendelian randomization to improve the design of Randomized trials. Cold Spring Harb Perspect Med 2021, 11(7). 10.1101/cshperspect.a040980 PMC8247560 33431510

[14] Davies NM, Holmes MV, Davey Smith G (2018). Reading mendelian randomisation studies: a guide, glossary, and checklist for clinicians. BMJ.

[15] VanderWeele TJ, Tchetgen Tchetgen EJ, Cornelis M, Kraft P (2014). Methodological challenges in mendelian randomization. Epidemiology.

[16] van de Vegte YJ, Said MA, Rienstra M, van der Harst P, Verweij N (2020). Genome-wide association studies and mendelian randomization analyses for leisure sedentary behaviours. Nat Commun.

[17] Dashti HS, Jones SE, Wood AR, Lane JM, van Hees VT, Wang H, Rhodes JA, Song Y, Patel K, Anderson SG (2019). Genome-wide association study identifies genetic loci for self-reported habitual sleep duration supported by accelerometer-derived estimates. Nat Commun.

[18] Jansen PR, Watanabe K, Stringer S, Skene N, Bryois J, Hammerschlag AR, de Leeuw CA, Benjamins JS, Munoz-Manchado AB, Nagel M (2019). Genome-wide analysis of insomnia in 1,331,010 individuals identifies new risk loci and functional pathways. Nat Genet.

[19] Dashti HS, Daghlas I, Lane JM, Huang Y, Udler MS, Wang H, Ollila HM, Jones SE, Kim J, Wood AR (2021). Genetic determinants of daytime napping and effects on cardiometabolic health. Nat Commun.

[20] Atkins JL, Jylhava J, Pedersen NL, Magnusson PK, Lu Y, Wang Y, Hagg S, Melzer D, Williams DM, Pilling LC (2021). A genome-wide association study of the frailty index highlights brain pathways in ageing. Aging Cell.

[21] Burgess S, Foley CN, Zuber V (2018). Inferring Causal relationships between Risk factors and outcomes from Genome-Wide Association Study Data. Annu Rev Genomics Hum Genet.

[22] Labrecque J, Swanson SA (2018). Understanding the assumptions underlying Instrumental variable analyses: a brief review of falsification strategies and related tools. Curr Epidemiol Rep.

[23] Burgess S, Thompson SG, Collaboration CCG (2011). Avoiding bias from weak instruments in mendelian randomization studies. Int J Epidemiol.

[24] Verbanck M, Chen CY, Neale B, Do R (2018). Detection of widespread horizontal pleiotropy in causal relationships inferred from mendelian randomization between complex traits and diseases. Nat Genet.

[25] Bowden J, Davey Smith G, Burgess S (2015). Mendelian randomization with invalid instruments: effect estimation and bias detection through Egger regression. Int J Epidemiol.

[26] Bowden J, Davey Smith G, Haycock PC, Burgess S (2016). Consistent estimation in mendelian randomization with some Invalid instruments using a weighted median estimator. Genet Epidemiol.

[27] Hemani G, Zheng J, Elsworth B, Wade KH, Haberland V, Baird D, Laurin C, Burgess S, Bowden J, Langdon R et al. The MR-Base platform supports systematic causal inference across the human phenome. Elife 2018, 7. 10.7554/eLife.34408 PMC5976434 29846171

[28] Burgess S, Thompson SG (2017). Interpreting findings from mendelian randomization using the MR-Egger method. Eur J Epidemiol.

[29] Kamat MA, Blackshaw JA, Young R, Surendran P, Burgess S, Danesh J, Butterworth AS, Staley JR (2019). PhenoScanner V2: an expanded tool for searching human genotype-phenotype associations. Bioinformatics.

[30] Yengo L, Sidorenko J, Kemper KE, Zheng Z, Wood AR, Weedon MN, Frayling TM, Hirschhorn J, Yang J, Visscher PM (2018). Meta-analysis of genome-wide association studies for height and body mass index in approximately 700000 individuals of European ancestry. Hum Mol Genet.

[31] Liu M, Jiang Y, Wedow R, Li Y, Brazel DM, Chen F, Datta G, Davila-Velderrain J, McGuire D, Tian C (2019). Association studies of up to 1.2 million individuals yield new insights into the genetic etiology of tobacco and alcohol use. Nat Genet.

[32] Ni G, Moser G, Genomics C, Wray NR, Lee SH, Schizophrenia Working Group of the Psychiatric (2018). Estimation of genetic correlation via linkage disequilibrium score regression and genomic restricted maximum likelihood. Am J Hum Genet.

[33] Skrivankova VW, Richmond RC, Woolf BAR, Yarmolinsky J, Davies NM, Swanson SA, VanderWeele TJ, Higgins JPT, Timpson NJ, Dimou N (2021). Strengthening the reporting of Observational studies in Epidemiology using mendelian randomization: the STROBE-MR Statement. JAMA.

[34] Ekelund U, Tarp J, Steene-Johannessen J, Hansen BH, Jefferis B, Fagerland MW, Whincup P, Diaz KM, Hooker SP, Chernofsky A (2019). Dose-response associations between accelerometry measured physical activity and sedentary time and all cause mortality: systematic review and harmonised meta-analysis. BMJ.

[35] Liu M, Hou T, Nkimbeng M, Li Y, Taylor JL, Sun X, Tang S, Szanton SL (2021). Associations between symptoms of pain, insomnia and depression, and frailty in older adults: a cross-sectional analysis of a cohort study. Int J Nurs Stud.

[36] Pan Z, Huang M, Huang J, Yao Z, Lin Z (2020). Association of napping and all-cause mortality and incident cardiovascular diseases: a dose-response meta analysis of cohort studies. Sleep Med.

[37] Burgess S, Davey Smith G, Davies NM, Dudbridge F, Gill D, Glymour MM, Hartwig FP, Kutalik Z, Holmes MV, Minelli C (2019). Guidelines for performing mendelian randomization investigations: update for summer 2023. Wellcome Open Res.

