# Supplementary material for: Causal associations between leisure sedentary behaviors and sleep status with frailty: insight from Mendelian randomization study
Source: BMC Geriatr. 2024 Feb 17;24:168. doi: 10.1186/s12877-024-04758-z (PMC10874533; doi:10.1186/s12877-024-04758-z)
Supplement: Supplementary file 1 — Supplementary Material 1 [file 12877_2024_4758_MOESM1_ESM.docx]

***Additional file 1***

**Causal associations between leisure sedentary behaviors and sleep status with frailty: insight from Mendelian randomization study**

Chuang Li ^1,2†^, Na Li ^1,2^^†^, Hailong Huang^1,2^, Yangyang Li ^1,2^, Yanyan Zhuang ^1,2*^

^1^Department of Obstetrics & Gynecology, Shengjing Hospital of China Medical University, Shenyang, China

^2^Key Laboratory of Maternal-Fetal Medicine of Liaoning Province, Shenyang, China

**Co-Correspondence:**

Yanyan Zhuang, Department of Obstetrics & Gynecology, Shengjing Hospital of China Medical University, Shenyang, China; Key Laboratory of Maternal-Fetal Medicine of Liaoning Province, Shenyang, China

E-mail: 19860109zyy@sina.com

^†^Chuang Li and Na Li contributed equally to this work

**STROBE-MR checklist of recommended items to address in reports of Mendelian randomization studies**^1^ ^2^

| **Item No.** | **Section** | **Checklist item** | **Page No.** | **Relevant text from manuscript** |
| --- | --- | --- | --- | --- |
| 1 | **TITLE and ABSTRACT** | Indicate Mendelian randomization (MR) as the study’s design in the title and/or the abstract if that is a main purpose of the study | 1 and 2 | Title: Causal associations between leisure sedentary behaviours and sleep status with frailty: insight from Mendelian randomization study  Abstract: Background: Observational studies have suggested that sedentary behaviors and sleep status are associated with frailty. However, it remains unclear whether these associations are causal.  Methods: Using summary statistics from genome-wide association studies, we evaluated the causal effect of modifiable risk factors, including leisure sedentary behaviors and sleep status on the frailty index (FI) using two-sample univariable and multivariable Mendelian randomization (MR) analyses. Genetic correlations were tested between the correlated traits.  Results: We identified potential causal associations between the time spent watching television (β = 0.26, 95% confidence interval [CI]: 0.21–0.31, P = 3.98e-25), sleep duration (β = -0.18, 95%CI: -0.26, -0.10; P = 6.04e-06), and daytime napping (β = 0.29, 95%CI: 0.18–0.41, P = 2.68e-07) and the FI based on the inverse-variance-weighted method. The estimates were consistent across robust and multivariate MR analyses. Linkage disequilibrium score regression detected a genetic correlation between the time spent watching television (Rg = 0.43, P = 6.46e-48), sleep duration (Rg = -0.20, P = 5.29e-10), and daytime napping (Rg = 0.25, P = 3.34e-21) and the FI.  Conclusions: Genetic predispositions to time spent watching television and daytime napping were positively associated with the FI, while sleep duration was negatively associated with the FI. Our findings offer key insights into factors influencing biological aging and suggest areas for interventions to promote healthy aging and slow down the aging process. |
|  | **INTRODUCTION** |  |  |  |
| 2 | **Background** | Explain the scientific background and rationale for the reported study. What is the exposure? Is a potential causal relationship between exposure and outcome plausible? Justify why MR is a helpful method to address the study question | 4 | Frailty is a complex clinical condition that is intricately linked to the aging process and is accompanied by a decline in physiological function across multiple organ systems, with increased susceptibility to stressors [1, 2]. As the global population ages, the prevalence of frailty increases proportionately [3]. Beyond its role as a noteworthy predictor of mortality among older individuals, frailty is implicated in a spectrum of adverse health outcomes, including falls, delirium, and disability [4-6]. In recent years, frailty has garnered considerable attention as a public health concern and tremendous global health challenge.  The roles of sedentary behavior and sleep status in the etiology of frailty have been extensively investigated [7-10]. However, owing to limitations in the quality of available evidence, as well as the presence of potential reverse causality and residual confounding factors, observational studies have been unable to establish causal associations. To address this issue, randomized controlled trials (RCTs) have been conducted to elucidate cause-and-effect relationships [11]. Nonetheless, RCTs are often resource-intensive in terms of finances and manpower, and certain interventions may not be feasible or approved for assessment. In this context, Mendelian randomization (MR) is effective for estimating the causal effects of exposures on outcomes [12, 13]. By employing genetic variants that are robustly associated with the exposure of interest as instrumental variables (IVs) randomly assigned at conception, MR studies mitigate the confounding and reverse causality biases that are inherent in conventional observational studies [14, 15]. |
| 3 | **Objectives** | State specific objectives clearly, including pre-specified causal hypotheses (if any). State that MR is a method that, under specific assumptions, intends to estimate causal effects | 5 | We conducted univariable and multivariable MR analyses to examine potential independent causal effects of leisure sedentary behaviors and sleep status on the frailty index (FI). Linkage disequilibrium score (LDSC) regression was used to investigate the genetic correlation between these causal traits. |
|  | **METHODS** |  |  |  |
| 4 | **Study design and data sources** | Present key elements of the study design early in the article. Consider including a table listing sources of data for all phases of the study. For each data source contributing to the analysis, describe the following: |  |  |
|  | a) | Setting: Describe the study design and the underlying population, if possible. Describe the setting, locations, and relevant dates, including periods of recruitment, exposure, follow-up, and data collection, when available. | 5 | We conducted univariable and multivariable MR analyses to examine potential independent causal effects of leisure sedentary behaviors and sleep status on the frailty index (FI). Linkage disequilibrium score (LDSC) regression was used to investigate the genetic correlation between these causal traits. |
|  | b) | Participants: Give the eligibility criteria, and the sources and methods of selection of participants. Report the sample size, and whether any power or sample size calculations were carried out prior to the main analysis | 5-7 | Data source of exposure  Watching television, computer usage, and driving were identified as three distinct categories of sedentary behaviors [16]. To ascertain the extent of sedentary time, the participants were asked a set of three questions during their initial visit. These inquiries encompassed the following: “On a typical day, how many hours do you spend watching television?,” “In a typical day, how many hours do you spend using the computer? (Do not include using a computer at work),” and “On a typical day, how many hours do you spend driving?.” The daily duration of these sedentary behaviors served as a measure of exposure assessment. In total, 408,815 individuals of European ancestry from the UK Biobank were included in this study. Mean daily reported leisure television watching was 2.8 h (±1.5), leisure computer use was 1.0 h (±1.2) and driving was 0.9 h (±1.0).  We acquired genetic instruments for sleep duration through a genome-wide association study (GWAS) conducted using the UK Biobank dataset [17]. Within the UK Biobank, the sleep duration assessment involved a specific inquiry: “How many hours of sleep do you get during every twenty-four hours (including naps)?” The results were scaled to per-hour increases in sleep duration. In this study, a total of 446,118 individuals of European descent was included, with females accounting for 54.1% of the total sample. The analysis revealed mean self-reported habitual sleep duration was 7.2 h (±1.1) per 24 h.  Genetic variants associated with snoring were obtained from the UK Biobank population [18]. During the assessment, participants were asked the following question: “Does your partner or a close relative or friend complain about your snoring?” Response options included “Yes,” “No,” “Don't know,” or “Prefer not to answer.” Individuals who responded with “Don't know” or “Prefer not to answer” were excluded from the dataset. Snoring data were available in 359,916 unrelated individuals of European descent after quality control. The prevalence of snoring in this sample was 37.3%.  Daytime napping is an uncontrollable sleep pattern. We acquired genetic variants associated with daytime napping from an extensive UK Biobank dataset comprising 452,633 participants [19]. During the assessment, the participants were asked, “Do you take a nap during the day?” Among the UK Biobank population, 38.2% and 5.3% of the respondents answered “sometimes” and “always,” respectively. Moreover, the average ages of the two groups were 58.5 (±7.8) years and 60.2 (±7.4) years, with females comprising 50.0% and 33.9% of each respective group.  Data source of outcome  We identified genetic variants associated with the FI using a GWAS meta-analysis. The GWAS included 164,610 participants from the UK Biobank and 10,616 from TwinGene [20]. The UK Biobank participants consisted of individuals of European descent, aged 60 to 70 years at baseline (mean 64.1, SD 2.8). The cohort included 84,819 females, accounting for 51.3% of the total participants. The TwinGene participants, a separate cohort comprising 10,616 individuals, were Swedish nationals aged 41 to 87 years (mean 58.3, SD 7.9), with 5,577 females (52.5%). The Rockwood FI, which is based on the deficit accumulation model, served as the outcome measure for frailty. Compliance with deficits was categorized using a score of 0 or 1 (with 0 indicating no deficit). The FI for each individual was calculated as the number of deficits divided by 49. A higher FI value indicates a greater degree of frailty. Our findings revealed that the mean proportions of deficits were 0.129 ± 0.075 and 0.121 ± 0.080, in UK Biobank and TwinGene participants, respectively.  The baseline characteristics of participants included in these above GWAS studies were presented in Additional file 1: Table S1. |
|  | c) | Describe measurement, quality control and selection of genetic variants | 7-8 | To ensure the validity of each instrumental variable (IV), three key assumptions must be met: (1) relevance assumption: robust association between the instrument and the exposure; (2) independence assumption: the genetic variant is not linked to confounding factors influencing the exposure-outcome relationship [21]; and (3) exclusion restriction assumption: instruments solely impact the outcome through the exposure variable [22]. To fulfill the first assumption of MR, we identified single nucleotide polymorphisms (SNPs) that achieved genome-wide significance (P < 5 × 10-8). From this SNP set, we exclusively retained independent instruments with the most significant P-values, considering pairwise linkage disequilibrium (LD) and removing SNPs with r2 ≥ 0.001. We further evaluated the first assumption by computing the F-statistic [23]. Subsequently, we excluded SNPs with an F-statistic < 10 to avoid weak IV biases. Genetic variants from diverse studies were combined regarding their effects and palindromic SNPs were excluded. Proxy SNPs (r2 ＞ 0.8) were used for instruments absent from the outcome dataset. The MR-Steiger filtering was also used to removes SNPs failing to explain significantly more variance in the exposure than in the outcome. To mitigate the bias arising from horizontal pleiotropy, we performed the Mendelian Randomization Pleiotropy RESidual Sum and Outlier (MR-PRESSO) outlier test, calculated the P value for each SNP, and excluded outlier SNPs [24]. The lead SNPs for the genetic instruments of exposure were presented in Additional file 2: Tables S2–S7. |
|  | d) | For each exposure, outcome, and other relevant variables, describe methods of assessment and diagnostic criteria for diseases | 5-7 | To ensure the validity of each instrumental variable (IV), three key assumptions must be met: (1) relevance assumption: robust association between the instrument and the exposure; (2) independence assumption: the genetic variant is not linked to confounding factors influencing the exposure-outcome relationship [21]; and (3) exclusion restriction assumption: instruments solely impact the outcome through the exposure variable [22]. To fulfill the first assumption of MR, we identified single nucleotide polymorphisms (SNPs) that achieved genome-wide significance (P < 5 × 10-8). From this SNP set, we exclusively retained independent instruments with the most significant P-values, considering pairwise linkage disequilibrium (LD) and removing SNPs with r2 ≥ 0.001. We further evaluated the first assumption by computing the F-statistic [23]. Subsequently, we excluded SNPs with an F-statistic < 10 to avoid weak IV biases. Genetic variants from diverse studies were combined regarding their effects and palindromic SNPs were excluded. Proxy SNPs (r2 ＞ 0.8) were used for instruments absent from the outcome dataset. The MR-Steiger filtering was also used to removes SNPs failing to explain significantly more variance in the exposure than in the outcome. To mitigate the bias arising from horizontal pleiotropy, we performed the Mendelian Randomization Pleiotropy RESidual Sum and Outlier (MR-PRESSO) outlier test, calculated the P value for each SNP, and excluded outlier SNPs [24]. The lead SNPs for the genetic instruments of exposure were presented in Additional file 2: Tables S2–S7. |
|  | e) | Provide details of ethics committee approval and participant informed consent, if relevant |  |  |
| 5 | **Assumptions** | Explicitly state the three core IV assumptions for the main analysis (relevance, independence and exclusion restriction) as well assumptions for any additional or sensitivity analysis | 7 | To ensure the validity of each instrumental variable (IV), three key assumptions must be met: (1) relevance assumption: robust association between the instrument and the exposure; (2) independence assumption: the genetic variant is not linked to confounding factors influencing the exposure-outcome relationship [21]; and (3) exclusion restriction assumption: instruments solely impact the outcome through the exposure variable [22]. |
| 6 | **Statistical methods: main analysis** | Describe statistical methods and statistics used |  |  |
|  | a) | Describe how quantitative variables were handled in the analyses (i.e., scale, units, model) | 5-7 | (1) Mean daily reported leisure television watching was 2.8 h (±1.5), leisure computer use was 1.0 h (±1.2) and driving was 0.9 h (±1.0). (2) The analysis revealed mean self-reported habitual sleep duration was 7.2 h (±1.1) per 24 h. (3) Snoring data were available in 359,916 unrelated individuals of European descent after quality control. The prevalence of snoring in this sample was 37.3%. (4) Among the UK Biobank population, 38.2% and 5.3% of the respondents answered “sometimes” and “always,” respectively. Moreover, the average ages of the two groups were 58.5 (±7.8) years and 60.2 (±7.4) years, with females comprising 50.0% and 33.9% of each respective group. (5) Our findings revealed that the mean proportions of deficits were 0.129 ± 0.075 and 0.121 ± 0.080, in UK Biobank and TwinGene participants, respectively. |
|  | b) | Describe how genetic variants were handled in the analyses and, if applicable, how their weights were selected | 8-9 | Univariable Mendelian randomization  We employed the inverse-variance-weighted (IVW) approach as the primary method for estimating causal effects, which allows calculation of the combined effect of all SNPs. Additionally, to ensure reliability and stability of the results, sensitivity analyses were conducted using MR-Egger [25], weighted median [26], and MR-PRESSO [24]. Heterogeneity in the main IVW and MR-Egger estimates was assessed by quantifying Cochran's Q statistic, where P > 0.1 indicated the absence of heterogeneity among the instrumental variables, enabling the disregard of its influence on causal effect estimation [27]. For cases in which heterogeneity was detected, the IVW (multiplicative random-effects) approach was used to calculate the effect size. The Egger model intercept was used for statistical assessment of pleiotropy, with a deviation of 0, suggesting the presence of directional pleiotropy [28].  Multivariable Mendelian randomization  To validate the second core assumption of MR, which entails assessing the absence of associations between the IVs and confounders, we conducted a thorough search of the PhenoScanner database [29]. This search aimed to identify any previously reported significant associations (P <5 × 10-8) between the instrument SNPs and potential confounding factors. Notably, the PhenoScanner search identified associations between the instruments and traits related to obesity, alcohol consumption, and smoking. Consequently, we specifically selected SNPs associated with body mass index (BMI) [30], drinks per week [31], and cigarettes per day [31] from the publicly available GWAS summary statistics to conduct multivariable MR (MVMR) analyses to account for indirect pathways that may introduce correlated pleiotropy. Causal effects were estimated using the MVMR-IVW and MVMR-Egger methods. Heterogeneity among the selected genetic variants was assessed using Cochran's Q statistic, and MVMR-Egger was employed to identify potential directional pleiotropy. |
|  | c) | Describe the MR estimator (e.g. two-stage least squares, Wald ratio) and related statistics. Detail the included covariates and, in case of two-sample MR, whether the same covariate set was used for adjustment in the two samples | 8-9 | Univariable Mendelian randomization  We employed the inverse-variance-weighted (IVW) approach as the primary method for estimating causal effects, which allows calculation of the combined effect of all SNPs. Additionally, to ensure reliability and stability of the results, sensitivity analyses were conducted using MR-Egger [25], weighted median [26], and MR-PRESSO [24]. Heterogeneity in the main IVW and MR-Egger estimates was assessed by quantifying Cochran's Q statistic, where P > 0.1 indicated the absence of heterogeneity among the instrumental variables, enabling the disregard of its influence on causal effect estimation [27]. For cases in which heterogeneity was detected, the IVW (multiplicative random-effects) approach was used to calculate the effect size. The Egger model intercept was used for statistical assessment of pleiotropy, with a deviation of 0, suggesting the presence of directional pleiotropy [28].  Multivariable Mendelian randomization  To validate the second core assumption of MR, which entails assessing the absence of associations between the IVs and confounders, we conducted a thorough search of the PhenoScanner database [29]. This search aimed to identify any previously reported significant associations (P <5 × 10-8) between the instrument SNPs and potential confounding factors. Notably, the PhenoScanner search identified associations between the instruments and traits related to obesity, alcohol consumption, and smoking. Consequently, we specifically selected SNPs associated with body mass index (BMI) [30], drinks per week [31], and cigarettes per day [31] from the publicly available GWAS summary statistics to conduct multivariable MR (MVMR) analyses to account for indirect pathways that may introduce correlated pleiotropy. Causal effects were estimated using the MVMR-IVW and MVMR-Egger methods. Heterogeneity among the selected genetic variants was assessed using Cochran's Q statistic, and MVMR-Egger was employed to identify potential directional pleiotropy. |
|  | d) | Explain how missing data were addressed |  |  |
|  | e) | If applicable, indicate how multiple testing was addressed |  |  |
| 7 | **Assessment of assumptions** | Describe any methods or prior knowledge used to assess the assumptions or justify their validity |  |  |
| 8 | **Sensitivity analyses and additional analyses** | Describe any sensitivity analyses or additional analyses performed (e.g. comparison of effect estimates from different approaches, independent replication, bias analytic techniques, validation of instruments, simulations) | 8-9 | Univariable Mendelian randomization  We employed the inverse-variance-weighted (IVW) approach as the primary method for estimating causal effects, which allows calculation of the combined effect of all SNPs. Additionally, to ensure reliability and stability of the results, sensitivity analyses were conducted using MR-Egger [25], weighted median [26], and MR-PRESSO [24]. Heterogeneity in the main IVW and MR-Egger estimates was assessed by quantifying Cochran's Q statistic, where P > 0.1 indicated the absence of heterogeneity among the instrumental variables, enabling the disregard of its influence on causal effect estimation [27]. For cases in which heterogeneity was detected, the IVW (multiplicative random-effects) approach was used to calculate the effect size. The Egger model intercept was used for statistical assessment of pleiotropy, with a deviation of 0, suggesting the presence of directional pleiotropy [28].  Multivariable Mendelian randomization  To validate the second core assumption of MR, which entails assessing the absence of associations between the IVs and confounders, we conducted a thorough search of the PhenoScanner database [29]. This search aimed to identify any previously reported significant associations (P <5 × 10-8) between the instrument SNPs and potential confounding factors. Notably, the PhenoScanner search identified associations between the instruments and traits related to obesity, alcohol consumption, and smoking. Consequently, we specifically selected SNPs associated with body mass index (BMI) [30], drinks per week [31], and cigarettes per day [31] from the publicly available GWAS summary statistics to conduct multivariable MR (MVMR) analyses to account for indirect pathways that may introduce correlated pleiotropy. Causal effects were estimated using the MVMR-IVW and MVMR-Egger methods. Heterogeneity among the selected genetic variants was assessed using Cochran's Q statistic, and MVMR-Egger was employed to identify potential directional pleiotropy. |
| 9 | **Software and pre-registration** |  |  |  |
|  | a) | Name statistical software and package(s), including version and settings used | 10 | All MR analyses were conducted using “TwoSampleMR,” “MRPRESSO,” “MendelianRandomization,” and “MVMR” in R software (version 4.0.2; the R Foundation for Statistical Computing, Vienna, Austria). |
|  | b) | State whether the study protocol and details were pre-registered (as well as when and where) |  |  |
|  | **RESULTS** |  |  |  |
| 10 | **Descriptive data** |  |  |  |
|  | a) | Report the numbers of individuals at each stage of included studies and reasons for exclusion. Consider use of a flow diagram |  |  |
|  | b) | Report summary statistics for phenotypic exposure(s), outcome(s), and other relevant variables (e.g. means, SDs, proportions) | 22 | Table 1 |
|  | c) | If the data sources include meta-analyses of previous studies, provide the assessments of heterogeneity across these studies |  |  |
|  | d) | For two-sample MR:  i.  Provide justification of the similarity of the genetic variant-exposure associations between the exposure and outcome samples  ii.  Provide information on the number of individuals who overlap between the exposure and outcome studies | 8 | The MR study found that the genetic predisposition to spend time watching television (β = 0.26, 95% confidence interval [CI]:0.21–0.31, P =3.98e-25) and daytime napping (β = 0.29, 95%CI: 0.18–0.41, P = 2.68e-07) were positively associated with the FI based on the IVW method, while the sleep duration (β = -0.18, 95%CI: -0.26, -0.10; P = 6.04e-06) was negatively associated with the FI (Table 2; Figure 1). All MR results were robust in several sensitivity analyses (Table 2). There was no obvious heterogeneity for the genetic variants of time spent on driving (all P-values for Cochran's Q > 0.1), whereas genetic instrumental variables of other exposures exhibited persistent heterogeneity (Additional file 2: Table S8). All P-values for the intercepts of MR-Egger tests were > 0.05 (Additional file 2: Table S9). |
| 11 | **Main results** |  |  |  |
|  | a) | Report the associations between genetic variant and exposure, and between genetic variant and outcome, preferably on an interpretable scale | 8-9 | The MR study found that the genetic predisposition to spend time watching television (β = 0.26, 95% confidence interval [CI]:0.21–0.31, P =3.98e-25) and daytime napping (β = 0.29, 95%CI: 0.18–0.41, P = 2.68e-07) were positively associated with the FI based on the IVW method, while the sleep duration (β = -0.18, 95%CI: -0.26, -0.10; P = 6.04e-06) was negatively associated with the FI (Table 2; Figure 1). All MR results were robust in several sensitivity analyses (Table 2). There was no obvious heterogeneity for the genetic variants of time spent on driving (all P-values for Cochran's Q > 0.1), whereas genetic instrumental variables of other exposures exhibited persistent heterogeneity (Additional file 2: Table S8). All P-values for the intercepts of MR-Egger tests were > 0.05 (Additional file 2: Table S9).  Multivariable MR analyses adjusting for confounders provided similar results and also suggested a positively causal effect of time spent on watching television (β = 0.27, 95% CI: 0.20 – 0.35, P = 1.19e-12) and daytime napping (β = 0.23, 95%CI: 0.10 – 0.36, P = 4.89e-04) and negative causal effect of sleep duration (β = -0.18, 95%CI: -0.27, -0.09; P = 1.55e-04) on the FI (Table 3). All directions and the statistical significance of the IVW results in MVMR were consistent with those of the MVMR-Egger sensitivity analysis, suggesting a low risk of bias due to horizontal pleiotropy (Table 3; Additional file 2: Table S9). The MVMR heterogeneity test validated sustained heterogeneity across the selected genetic variants (Additional file 2: Table S10). |
|  | b) | Report MR estimates of the relationship between exposure and outcome, and the measures of uncertainty from the MR analysis, on an interpretable scale, such as odds ratio or relative risk per SD difference | 8-9 | The MR study found that the genetic predisposition to spend time watching television (β = 0.26, 95% confidence interval [CI]:0.21–0.31, P =3.98e-25) and daytime napping (β = 0.29, 95%CI: 0.18–0.41, P = 2.68e-07) were positively associated with the FI based on the IVW method, while the sleep duration (β = -0.18, 95%CI: -0.26, -0.10; P = 6.04e-06) was negatively associated with the FI (Table 2; Figure 1). All MR results were robust in several sensitivity analyses (Table 2). There was no obvious heterogeneity for the genetic variants of time spent on driving (all P-values for Cochran's Q > 0.1), whereas genetic instrumental variables of other exposures exhibited persistent heterogeneity (Additional file 2: Table S8). All P-values for the intercepts of MR-Egger tests were > 0.05 (Additional file 2: Table S9).  Multivariable MR analyses adjusting for confounders provided similar results and also suggested a positively causal effect of time spent on watching television (β = 0.27, 95% CI: 0.20 – 0.35, P = 1.19e-12) and daytime napping (β = 0.23, 95%CI: 0.10 – 0.36, P = 4.89e-04) and negative causal effect of sleep duration (β = -0.18, 95%CI: -0.27, -0.09; P = 1.55e-04) on the FI (Table 3). All directions and the statistical significance of the IVW results in MVMR were consistent with those of the MVMR-Egger sensitivity analysis, suggesting a low risk of bias due to horizontal pleiotropy (Table 3; Additional file 2: Table S9). The MVMR heterogeneity test validated sustained heterogeneity across the selected genetic variants (Additional file 2: Table S10). |
|  | c) | If relevant, consider translating estimates of relative risk into absolute risk for a meaningful time period |  |  |
|  | d) | Consider plots to visualize results (e.g. forest plot, scatterplot of associations between genetic variants and outcome versus between genetic variants and exposure) |  | Figure 1 |
| 12 | **Assessment of assumptions** |  |  |  |
|  | a) | Report the assessment of the validity of the assumptions | 8-9 | The MR study found that the genetic predisposition to spend time watching television (β = 0.26, 95% confidence interval [CI]:0.21–0.31, P =3.98e-25) and daytime napping (β = 0.29, 95%CI: 0.18–0.41, P = 2.68e-07) were positively associated with the FI based on the IVW method, while the sleep duration (β = -0.18, 95%CI: -0.26, -0.10; P = 6.04e-06) was negatively associated with the FI (Table 2; Figure 1). All MR results were robust in several sensitivity analyses (Table 2). There was no obvious heterogeneity for the genetic variants of time spent on driving (all P-values for Cochran's Q > 0.1), whereas genetic instrumental variables of other exposures exhibited persistent heterogeneity (Additional file 2: Table S8). All P-values for the intercepts of MR-Egger tests were > 0.05 (Additional file 2: Table S9).  Multivariable MR analyses adjusting for confounders provided similar results and also suggested a positively causal effect of time spent on watching television (β = 0.27, 95% CI: 0.20 – 0.35, P = 1.19e-12) and daytime napping (β = 0.23, 95%CI: 0.10 – 0.36, P = 4.89e-04) and negative causal effect of sleep duration (β = -0.18, 95%CI: -0.27, -0.09; P = 1.55e-04) on the FI (Table 3). All directions and the statistical significance of the IVW results in MVMR were consistent with those of the MVMR-Egger sensitivity analysis, suggesting a low risk of bias due to horizontal pleiotropy (Table 3; Additional file 2: Table S9). The MVMR heterogeneity test validated sustained heterogeneity across the selected genetic variants (Additional file 2: Table S10). |
|  | b) | Report any additional statistics (e.g., assessments of heterogeneity across genetic variants, such as *I^2^*, Q statistic or E-value) | 8-9 | The MR study found that the genetic predisposition to spend time watching television (β = 0.26, 95% confidence interval [CI]:0.21–0.31, P =3.98e-25) and daytime napping (β = 0.29, 95%CI: 0.18–0.41, P = 2.68e-07) were positively associated with the FI based on the IVW method, while the sleep duration (β = -0.18, 95%CI: -0.26, -0.10; P = 6.04e-06) was negatively associated with the FI (Table 2; Figure 1). All MR results were robust in several sensitivity analyses (Table 2). There was no obvious heterogeneity for the genetic variants of time spent on driving (all P-values for Cochran's Q > 0.1), whereas genetic instrumental variables of other exposures exhibited persistent heterogeneity (Additional file 2: Table S8). All P-values for the intercepts of MR-Egger tests were > 0.05 (Additional file 2: Table S9).  Multivariable MR analyses adjusting for confounders provided similar results and also suggested a positively causal effect of time spent on watching television (β = 0.27, 95% CI: 0.20 – 0.35, P = 1.19e-12) and daytime napping (β = 0.23, 95%CI: 0.10 – 0.36, P = 4.89e-04) and negative causal effect of sleep duration (β = -0.18, 95%CI: -0.27, -0.09; P = 1.55e-04) on the FI (Table 3). All directions and the statistical significance of the IVW results in MVMR were consistent with those of the MVMR-Egger sensitivity analysis, suggesting a low risk of bias due to horizontal pleiotropy (Table 3; Additional file 2: Table S9). The MVMR heterogeneity test validated sustained heterogeneity across the selected genetic variants (Additional file 2: Table S10). |
| 13 | **Sensitivity analyses and additional analyses** |  |  |  |
|  | a) | Report any sensitivity analyses to assess the robustness of the main results to violations of the assumptions | 11 | Table 1 |
|  | b) | Report results from other sensitivity analyses or additional analyses | 11 | Table 1, Table S8-S10 |
|  | c) | Report any assessment of direction of causal relationship (e.g., bidirectional MR) |  |  |
|  | d) | When relevant, report and compare with estimates from non-MR analyses |  |  |
|  | e) | Consider additional plots to visualize results (e.g., leave-one-out analyses) |  | Figure 1 |
|  | **DISCUSSION** |  |  |  |
| 14 | **Key results** | Summarize key results with reference to study objectives | 11 | This MR study investigated the potential causal relationships between the leisure sedentary behaviors, sleep status and the FI. We found that time spent watching television, sleep duration, and daytime napping were causally associated with the FI. Multiple sensitivity analyses confirmed the robustness of these causal relationships. MVMR analyses demonstrated independent causal effects of watching television, sleep duration, and daytime napping on the FI, after adjustments for other confounders. We observed a significant genetic correlation between the time spent watching television, sleep duration, daytime napping, and the FI based on the LDSC regression. |
| 15 | **Limitations** | Discuss limitations of the study, taking into account the validity of the IV assumptions, other sources of potential bias, and imprecision. Discuss both direction and magnitude of any potential bias and any efforts to address them | 13 | First, our analysis assumed a linear association between risk factors and outcomes. Although the estimates reflect the presence and direction of the population-averaged causal effect, quantitative interpretations may be misleading if the actual relationship is nonlinear [37]. Second, genetic associations were derived from data of European populations, and caution should be exercised when generalizing these findings to other ethnic groups. Third, the MR analysis estimates were formulated to evaluate the causal impact of long-term exposure on outcomes and, therefore, may not always align precisely with clinical observations. Fourth, while we attempted to strictly adhere to the STROBE-MR guidelines, we were unable to provide all the recommended items because of the restricted information accessible from the utilized database. |
| 16 | **Interpretation** |  |  |  |
|  | a) | Meaning: Give a cautious overall interpretation of results in the context of their limitations and in comparison with other studies | 11-12 | Population-based investigations have consistently demonstrated that sedentary behavior contributes to adverse health outcomes. A meta-analysis revealed that reduced sedentary duration is significantly associated with a decreased risk of premature mortality, particularly among middle-aged and older adults, exhibiting a nonlinear dose-response pattern [34]. Considering the findings of our research and those from previous studies, reducing sedentary duration during leisure activities has potential benefits for mitigating the aging process.  Significant causal associations were observed between certain sleep status traits and the FI. Numerous studies have reported a relationship between sleep and aging. A study conducted in a Chinese population comprising 23,847 individuals revealed that maintaining a comprehensive and healthy sleep pattern was positively linked to a reduced risk of worsening frailty and an increased likelihood of improving frailty [10]. Another cross-sectional study utilizing data from the National Health and Aging Trends Study, identified difficulty initiating sleep as an independent risk factor for frailty [35]. Furthermore, a meta-analysis involving 313,651 participants from 20 cohort studies demonstrated that prolonged napping is associated with a higher risk of all-cause mortality [36]. After adjusting for relevant factors, we provide compelling evidence supporting significant causal relationships between sleep duration and daytime napping with the FI. |
|  | b) | Mechanism: Discuss underlying biological mechanisms that could drive a potential causal relationship between the investigated exposure and the outcome, and whether the gene-environment equivalence assumption is reasonable. Use causal language carefully, clarifying that IV estimates may provide causal effects only under certain assumptions |  |  |
|  | c) | Clinical relevance: Discuss whether the results have clinical or public policy relevance, and to what extent they inform effect sizes of possible interventions | 13 | In summary, we utilized MR techniques to provide valuable quantitative data on modifiable risk factors that causally influence the aging process. Understanding the causal effects of these risk factors on frailty holds considerable promise for elucidating the underlying mechanisms of the aging process and establishing potential strategies for preventing age-related diseases and promoting healthy aging. Notably, significant effects were observed for the time spent watching television, sleep duration, and daytime napping. These findings provide crucial insights into the determinants of biological aging and highlight potential areas for intervention to promote healthy longevity and attenuate the rate of biological aging. |
| 17 | **Generalizability** | Discuss the generalizability of the study results (a) to other populations, (b) across other exposure periods/timings, and (c) across other levels of exposure | 13 | In summary, we utilized MR techniques to provide valuable quantitative data on modifiable risk factors that causally influence the aging process. Understanding the causal effects of these risk factors on frailty holds considerable promise for elucidating the underlying mechanisms of the aging process and establishing potential strategies for preventing age-related diseases and promoting healthy aging. Notably, significant effects were observed for the time spent watching television, sleep duration, and daytime napping. These findings provide crucial insights into the determinants of biological aging and highlight potential areas for intervention to promote healthy longevity and attenuate the rate of biological aging. |
|  | **OTHER INFORMATION** |  |  |  |
| 18 | **Funding** | Describe sources of funding and the role of funders in the present study and, if applicable, sources of funding for the databases and original study or studies on which the present study is based | 14 | This work was supported by grants from the Applied basic research program for young scholars of Liaoning Province (no.2023JH2/101600023). |
| 19 | **Data and data sharing** | Provide the data used to perform all analyses or report where and how the data can be accessed, and reference these sources in the article. Provide the statistical code needed to reproduce the results in the article, or report whether the code is publicly accessible and if so, where | 14 | UK Biobank data can be found at http://www.nealelab.is/uk-biobank/. Data from meta-GWASs can be found in the raw publications. The PubMed or database ID of each GWAS is shown in Table 1. |
| 20 | **Conflicts of Interest** | All authors should declare all potential conflicts of interest | 15 | The authors declare that they have no conflicts of interest. |

This checklist is copyrighted by the Equator Network under the Creative Commons Attribution 3.0 Unported (CC BY 3.0) license.

1. Skrivankova VW, Richmond RC, Woolf BAR, Yarmolinsky J, Davies NM, Swanson SA, et al. Strengthening the Reporting of Observational Studies in Epidemiology using Mendelian Randomization (STROBE-MR) Statement. JAMA. 2021;under review.

2. Skrivankova VW, Richmond RC, Woolf BAR, Davies NM, Swanson SA, VanderWeele TJ, et al. Strengthening the Reporting of Observational Studies in Epidemiology using Mendelian Randomisation (STROBE-MR): Explanation and Elaboration. BMJ. 2021;375:n2233.
